# Supplementary material for: Quantum Kernel Learning for Small Dataset Modeling in Semiconductor Fabrication: Application to Ohmic Contact
Source: Adv Sci (Weinh). 2025 Jun 23;12(35):e06213. doi: 10.1002/advs.202506213 (PMC12462921; doi:10.1002/advs.202506213)
Supplement: Supplementary file 1 — Supporting Information [file ADVS-12-e06213-s001.docx]

**Quantum Kernel Learning for Small Dataset Modeling in Semiconductor Fabrication: Application to Ohmic Contact**

Zeheng Wang**^1, 2, †^**, Fangzhou Wang^3^, Liang Li^4^, Zirui Wang^5^, Timothy van der Laan**^2^**, Ross C. C. Leon^6^, Jing-Kai Huang^7^, and Muhammad Usman**^1,8^**

**^1^** Data61, CSIRO, Clayton, VIC 3168, Australia

**^2^** Manufacturing, CSIRO, West Lindfield, NSW 2070, Australia

^3^ Songshan Lake Materials Laboratory, Dongguan, 523808, China

^4^ Academy for Advanced Interdisciplinary Studies, Peking University, Beijing 100871, China

^5^ School of Integrated Circuits, Peking University, Beijing 100871, China

^6^ Quantum Motion Ltd, London N7 9HJ, United Kingdom

^7^ Department of Systems Engineering, City University of Hong Kong, Hong Kong 999077, China

^8^ School of Physics, The University of Melbourne, Parkville, VIC 3010, Australia

**† Corresponding author. Email: zenwang@outlook.com**

Keywords: Quantum Machine Learning, Quantum Kernel, Semiconductor Device, Fabrication


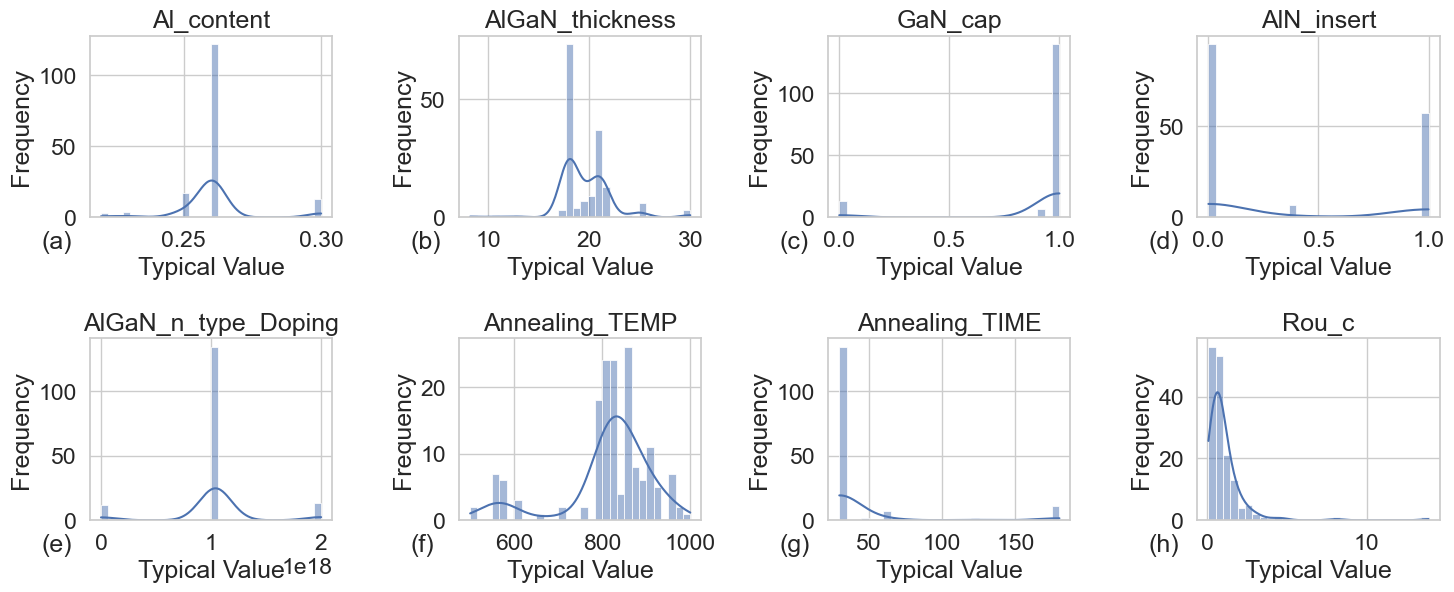


**SF. 1.** The distribution of the dataset’s key features and label.

| **Model** | **Model Name** | **Parameter Settings** |
| --- | --- | --- |
| EN | ElasticNet | alpha = 0.1, l1_ratio = 0.5 |
| SVM | Support Vector Regression (SVR) | kernel = 'linear', C = 1.0, gamma = 'scale' |
| DT | Decision Tree Regressor | max_depth = 10, min_samples_split = 5 |
| GB | Gradient Boosting Regressor | n_estimators = 150, learning_rate = 0.05, max_depth = 5 |
| XGB | XGBoost Regressor | default (max_depth = 6, learning_rate = 0.3, n_estimators = 100) |
| AB | AdaBoost Regressor | n_estimators = 100 |
| DL | Custom Deep Learning Model | 5-layer MLP: [input, 160, 80, 40, 20, 1] with Sigmoid → ReLU → ReLU → Tanh activations, 0.5 dropout after each layer |

**SF. Table 1.** The configuration of the CML models.

**SF. Table 2.** Recent progress of QML modeling.

| **Task** | **Score** | **QML alg.** | **qubits** | **QML score** | **CML alg.** | **Best CML score** | **Datasets** | **Ref.** |
| --- | --- | --- | --- | --- | --- | --- | --- | --- |
| Class. | ACC | Single qubit CNN | 1 | 0.946 | N.A | N.A | MNIST | ^[1]^ |
|  |  | Single qubit CNN | 1 | 0.895 | N.A | N.A | FMNIST |  |
|  |  | Single qubit CNN | 1 | 0.825 | N.A | N.A | Face Recognition |  |
| Class. | ACC | QNNN | 16 | 0.92 | MLP | 0.5 | Quantum synthesis data | ^[2]^ |
|  |  | QNNN | 13 | 0.7 | MLP | 0.85 | Wine |  |
|  |  | QCNN | 4 | 0.93 | CNN | 0.96 | MNIST |  |
|  |  | QNN | N/A | 0.8 | Classical NN | 0.88 | NISQ device |  |
| Reg. | Sum-Rate | QNN | 4 | 0.932 | Classical NN | 0.933 | Tetris | ^[3]^ |
|  |  | QNN | 4 | 0.9235 | Classical NN | 0.9134 | Tetris |  |
|  |  | QNN | 4 | 0.9191 | Classical NN | 0.9135 | Tetris |  |
|  |  | QCNN | 2 | 0.9262 | Classical NN | 0.9103 | Tetris |  |
|  |  | QCNN | 2 | 0.9149 | Classical NN | 0.9135 | Tetris |  |
|  |  | QCNN | 2 | 0.9223 | Classical NN | 0.8988 | Tetris |  |
|  |  | QNN | 4 | 0.9202 | Classical NN | 0.9189 | FakeLima |  |
|  |  | QNN | 4 | 0.9189 | Classical NN | 0.9189 | FakeCasablanca |  |
|  |  | QKSAN | 4 | 0.98 | N/A | N/A | Fashion MNIST |  |
|  |  | QKSAN | 4 | 0.99 | N/A | N/A | MNIST |  |
|  |  | QKSAN | 4 | 0.985 | N/A | N/A | Fashion MNIST |  |
| Class. | ACC | QSAN | 18 | 0.867 | CNN | 0.51 | CIFAR-10 | ^[4]^ |
|  |  | QSAN | 18 | 0.867 | MLP | 0.5 | CIFAR-10 |  |
|  |  | QSAN | 18 | 0.867 | RNN | 0.622 | CIFAR-10 |  |
| Reg. | RMSE | QGRU | 42 | 1.775 | LSTM-ARO | 4.768 | AAPL | ^[5]^ |
|  |  | QLSTM | 63 | 1.78 | LSTM-ARO | 4.768 | AAPL |  |
|  |  | QGRU | 42 | 1.775 | LSTM-GA | 4.91 | AAPL |  |
|  |  | QLSTM | 63 | 1.78 | LSTM-GA | 4.91 | AAPL |  |
|  |  | QGRU | 42 | 1.752 | LSTM-ARO | 5.802 | BA |  |
|  |  | QLSTM | 63 | 1.756 | LSTM-ARO | 5.802 | BA |  |
|  |  | QGRU | 42 | 1.752 | LSTM-GA | 6.342 | BA |  |
|  |  | QLSTM | 63 | 1.756 | LSTM-GA | 6.342 | BA |  |
| Class. | ACC | QA-HFNN | 1 | 0.84 | FDNN | 0.838 | Dirty-MNIST | ^[6]^ |
|  |  | QA-HFNN | 1 | 0.84 | ResNet18 | 0.839 | Dirty-MNIST |  |
|  |  | QA-HFNN | 1 | 0.84 | ResNet50 | 0.834 | Dirty-MNIST |  |
|  |  | QA-HFNN | 1 | 0.84 | Fuzzy pattern | 0.71 | Dirty-MNIST |  |
| **Reg.** | **MAE** | **QKAR** | **5** | **0.338** | **7 CML models** | **0.404** | **GaN contact fab dataset** | **This work** |
|  | **MSE** | **QKAR** | **5** | **0.218** |  | **0.273** | **GaN contact fab dataset** |  |
|  | **RMSE** | **QKAR** | **5** | **0.437** |  | **0.479** | **GaN contact fab dataset** |  |

**Supporting Algorithm 1**: Metal Layer Parsing and Data Preprocessing for AlGaN/GaN Experiment (Encoding the raw data into a dataset.)

**Input**: CSV dataset containing experimental parameters, including metal layer compositions.

**Output**: Processed dataset with one-hot encoded metal layers, thickness vectors, and imputed missing values.

BEGIN

# Step 1: Read CSV File

Load dataset df from file_path

# Step 2: Parse Metal Layers and Thicknesses

DEFINE FUNCTION parse_metal_layers(layer_str):

IF layer_str contains '(' and ')':

SPLIT metals and thicknesses at '('

metals ← SPLIT metals at '/'

thicknesses ← REMOVE 'nm', '(', ')' from thicknesses, then SPLIT at '/'

CONVERT thicknesses to float list

ELSE:

metals ← SPLIT layer_str at '/'

thicknesses ← LIST of zeros with length of metals

RETURN metals, thicknesses

df['Metals'] ← APPLY parse_metal_layers() to df['Metal_layers'] (extract metals)

df['Thicknesses'] ← APPLY parse_metal_layers() to df['Metal_layers'] (extract thicknesses)

# Step 3: One-Hot Encoding of Metal Layers

unique_metals ← SORTED(UNIQUE metals in df['Metals'])

INITIALIZE OneHotEncoder metal_encoder

metal_encoded ← APPLY metal_encoder to df['Metals']

metal_encoded_df ← CONVERT metal_encoded to DataFrame

df ← CONCATENATE df with metal_encoded_df

# Step 4: Construct Metal Thickness Vectors

DEFINE FUNCTION get_thickness_vector(metals, thicknesses, unique_metals):

thickness_vector ← []

FOR each metal in unique_metals:

IF metal IN metals:

index ← metals.index(metal)

APPEND thicknesses[index] to thickness_vector

ELSE:

APPEND 0.0 to thickness_vector

RETURN thickness_vector

df_thickness_vectors ← APPLY get_thickness_vector() to df rows

thickness_df ← CONVERT df_thickness_vectors to DataFrame

df ← CONCATENATE df with thickness_df

# Step 5: Data Cleaning and Imputation

REMOVE columns 'Metal_layers', 'Metals', 'Thicknesses'

columns_to_impute ← ['Al_content', 'AlGaN_thickness', 'GaN_cap', 'AlN_insert', 'AlGaN_n_type_Doping', 'Annealing_TEMP', 'Annealing_TIME']

APPLY mean imputation to columns_to_impute

# Step 6: Save Processed Data

SAVE df to output_file_path

PRINT "Processed data saved to output_file_path"

END

**Supporting Algorithm 2:** Standardization and Principal Component Analysis (PCA) on Processed AlGaN Data

**Input**: CSV dataset containing processed experimental data (data/processed_AlGaN-exp.csv).
**Output**: Standardized dataset and PCA-transformed dataset with n_components principal components.

CLASS database:

BEGIN CONSTRUCTOR __init__()

# Step 1: Read Processed CSV Data

Load dataset data from 'data/processed_AlGaN-exp.csv'

# Step 2: Extract Features and Target Variable

X_raw ← REMOVE column 'Rou_c' from data

y_raw ← EXTRACT column 'Rou_c' from data

# Step 3: Standardization using StandardScaler

INITIALIZE StandardScaler std_scaler

X_scaled_np ← APPLY std_scaler to X_raw (standardize features)

# Step 4: Convert Standardized Data to DataFrame and Tensor Format

X_scaled_pd ← CONVERT X_scaled_np to Pandas DataFrame with original column names

X ← CONVERT X_scaled_np to Torch tensor (dtype=torch.float32)

y ← CONVERT y_raw to Torch tensor (dtype=torch.float32, reshape to column vector)

# Step 5: Store Processed Data in Class Attributes

SET self.X_pdframe ← X_scaled_pd

SET self.y_df ← y_raw

END CONSTRUCTOR

FUNCTION apply_pca(n_components)

# Step 6: Perform PCA Dimensionality Reduction

INITIALIZE PCA with n_components

X_pca ← APPLY PCA to self.X_pdframe

# Step 7: Convert PCA Output to DataFrame

X_pca_df ← CONVERT X_pca to Pandas DataFrame

SET column names as ['PC1', 'PC2', ..., 'PCn_components']

# Step 8: Return PCA-Transformed Data

RETURN X_pca_df

END FUNCTION

END CLASS


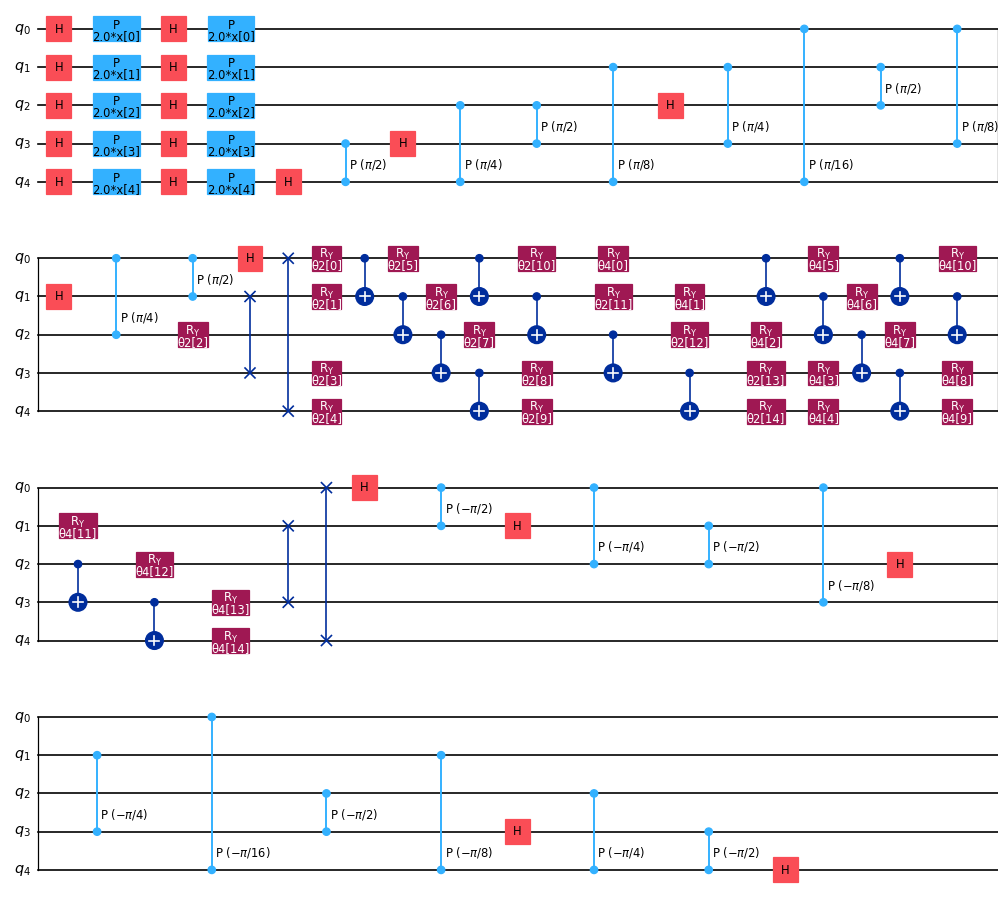


**SF. 2.** A conventional quantum neural network (QNN) containing sufficient quantum entanglement between qubits for benchmarking the modeling performance of the proposed QKAR.


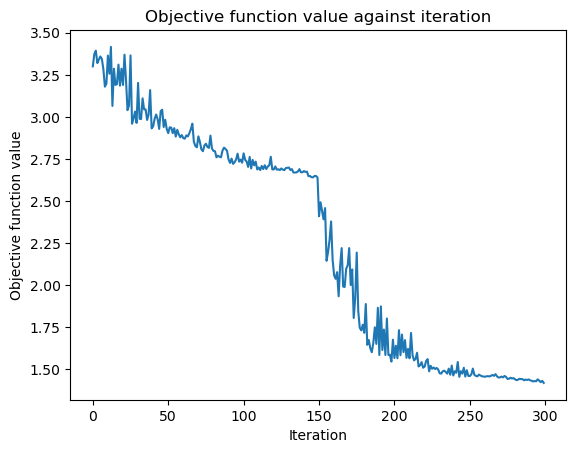


**SF. 3.** raining loss of the conventional quantum neural network (QNN). This result serves as a preliminary demonstration and should not be overinterpreted. Further investigation is required to assess the QNN's potential comprehensively. Nevertheless, it is worth noting that recent studies have shown many QNNs can be formally expressed as equivalent quantum kernel models^[7]^.


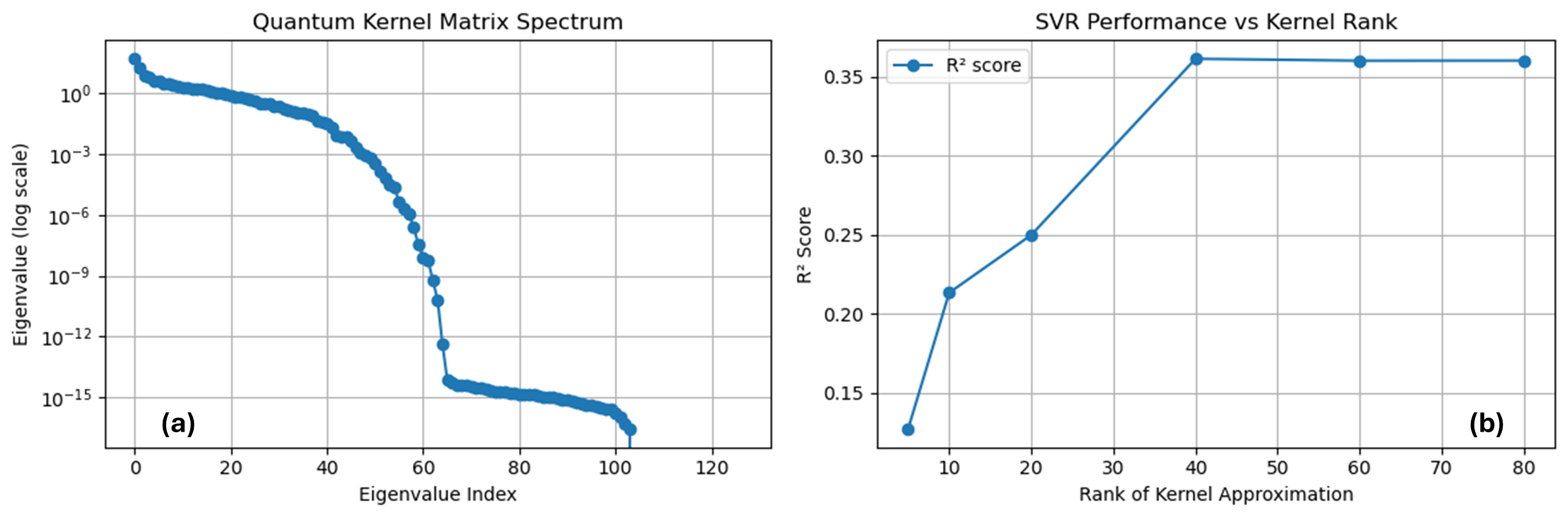


**SF. 4.** The quantum kernel matrix's properties and its performance in an SVR task (constructed using the Pauli-Z-QKA embedding and a randomly split training set). The results demonstrate that the kernel method is effective, and the model has not collapsed. (a) the eigenvalue spectrum of the kernel matrix, with values decaying rapidly from $10⁰$ to ${10}^{15}$, indicating a potentially high condition number (~${10}^{15}$). This suggests a certain level of numerical instability, particularly under finite shots or noise, which could amplify errors in matrix operations. However, (b) demonstrates that a low-rank approximation (up to rank 40) yields a stable SVR performance, with the $R^{2}$ score plateauing at ~0.35. This indicates that the dominant eigenvalues capture the essential data structure, and the high condition number—driven by near-zero eigenvalues—does not significantly impact performance in this low-rank setting.

- **References of Supporting Information**

[1] P. Easom-McCaldin, A. Bouridane, A. Belatreche, R. Jiang, S. Al-Maadeed, *IEEE Trans. Neural Netw. Learn. Syst.* **2024**, *35*, 1472.

[2] Y. Qian, X. Wang, Y. Du, X. Wu, D. Tao, *IEEE Trans. Neural Netw. Learn. Syst.* **2024**, *35*, 5603.

[3] J. Liu, K. H. Lim, K. L. Wood, W. Huang, C. Guo, H.-L. Huang, *Sci. China Phys. Mech. Astron.* **2021**, *64*, 290311.

[4] J. Shi, R.-X. Zhao, W. Wang, S. Zhang, X. Li, *IEEE Trans. Neural Netw. Learn. Syst.* **2024**, 1.

[5] Y. Li, Z. Wang, R. Xing, C. Shao, S. Shi, J. Li, G. Zhong, Y. Gu, *IEEE Trans. Pattern Anal. Mach. Intell.* **2025**, *47*, 2493.

[6] S. Wu, R. Li, Y. Song, S. Qin, Q. Wen, F. Gao, *IEEE Trans. Fuzzy Syst.* **2025**, *33*, 491.

[7] M. Schuld, *Supervised quantum machine learning models are kernel methods*, arXiv **2021**.
